# *McAPRR2*: The Key Regulator of Domesticated Pericarp Color in Bitter Gourd

**DOI:** 10.3390/plants12203585

**Published:** 2023-10-16

**Authors:** Shouwei Tian, Jingjing Yang, Yiqian Fu, Xiaofei Zhang, Jian Zhang, Hong Zhao, Qi Hu, Pangyuan Liu, Weiming He, Xiangyang Han, Changlong Wen

**Affiliations:** Beijing Key Laboratory of Vegetable Germplasms Improvement, Key Laboratory of Biology and Genetics Improvement of Horticultural Crops (North China), State Key Laboratory of Vegetable Biobreeding, National Engineering Research Center for Vegetables, Beijing Vegetable Research Center, Beijing Academy of Agriculture and Forestry Science, Beijing 100097, Chinayangjingjing@nercv.org (J.Y.); zhangjian@nercv.org (J.Z.); zhaohong@nercv.org (H.Z.);

**Keywords:** *Momordica charantia*, *McAPRR2*, pericarp color, haplotype, domestication

## Abstract

Pericarp color is a crucial commercial trait influencing consumer preferences for bitter gourds. However, until now, the gene responsible for this trait has remained unidentified. In this study, we identified a gene (*McAPRR2*) controlling pericarp color via a genome-wide association study (GWAS) utilizing the resequencing data of 106 bitter gourd accessions. *McAPRR2* exhibits three primary haplotypes: Hap1 is a wild type with a green pericarp, Hap2 is a SA (South Asian) and SEA (Southeast Asia) type with a green pericarp, and Hap3 is primarily a SEA type with a light green pericarp. The *McAPRR2* haplotype is significantly correlated with both pericarp color and ecological type. Importantly, *McAPRR2* with the light green pericarp demonstrated premature termination due to a 15 bp sequence insertion. The phylogenetic tree clustered according to pericarp color and ecological type, using SNPs located in the *McAPRR2* gene and its promoter. High π_wild/SEA_ and π_SA/SEA_ values indicate high nucleotide diversity between wild and SEA types and between SA and SEA types in the *McAPRR2* gene. The haplotypes, phylogenetic tree, and nucleotide diversity of *McAPRR2* suggest that *McAPRR2* has undergone domestication selection. This study identifies *McAPRR2* as the key gene determining pericarp color in bitter gourds and introduces a novel insight that *McAPRR2* is subject to domestication selection.

## 1. Introduction

Bitter gourd (*Momordica charantia*), known for its distinctive bitter taste, is a vegetable that is widely cultivated in tropical and subtropical Asia. It has been extensively used for health preservation due to its broad pharmacological properties, notably its anti-diabetic, anti-cancer, and anti-HIV effects [1,2,3,4]. Bitter gourd is native to tropical Africa [5] and Madagascar [6]. Study of the domestication region of bitter gourd is contentious. Earlier studies reported its domestication in Eastern Asia, possibly eastern India and southern China [7,8]. Schaefer and Renner [5] reported that bitter gourd from tropical Africa dispersed to Asia approximately 19 million years ago. Matsumura et al. [9] suggest the initial domestication of bitter gourd occurred around 6000 years ago, followed by the divergence of Southeast Asian cultivars around 800 years ago. According to Cui et al. [10], *M*. *charantia* var. *muricata* and *charantia* var. *charantia* split roughly 1.9 million years ago, while the separation between *muricata* and *charantia* happened once again about 6000 years ago. Based on these parallel studies, Renner [11] raised a new insight that bitter gourd from Africa expanded to Southeast Asia and was domesticated there.

Over the course of long-term evolution, plants have undergone domestication and selective breeding to adapt to human needs and environmental changes, leading to significant differences in many aspects between modern cultivated varieties and their wild counterparts. These traits include but are not limited to erect plant architecture [12], fruit weight [13], seed shattering [14], and pericarp color [15]. Several studies indicate that during the process of domestication and selection, the selective forces on certain genes are closely associated with the adaptability of the species to specific geographic locations [16,17]. For example, in rice, a red pericarp color is prevalent in wild populations and early landraces, whereas modern cultivars predominantly display a white pericarp color. Similarly, in bitter gourd, individuals from different regions show different pericarp colors; those found in South Asia typically present a green pericarp color, while those predominantly found in Southeast Asia often display a white or light green pericarp color [18]. This indicates that pericarp color may have been subjected to selective pressures during the process of domestication.

Pericarp color is primarily determined by the content of chlorophyll, carotene, anthocyanin, and flavonoids in the skin, and the genes regulating the pericarp color have been extensively investigated within the *Cucurbitaceae* family. In cucumber, the white pericarp trait is regulated by an *APRR2* gene (two-component response regulator-like *Csa3G904140*), where a single G insertion leads to early translation termination. Meanwhile, the light green pericarp trait is controlled by the *Csa6G133820* gene [19,20]. In watermelon, Oren et al. [21] identified an SNP of G to C mutation in the intron of *ClAPRR2* (*CICG09G012330*) gene leading to premature translation termination in the light green watermelon, while Li et al. [22] discovered a C to G nonsynonymous SNP mutation in the coding region of *ClCG08G017810* (2-phytyl-1,4-beta-naphthoquinone methyltransferase protein) responsible for the pericarp color from dark green to light green. In melon, two studies concluded that the *CmAPRR2* (*MELO3C003375*) controlled a pair of dark and light green pericarp traits. A mutation of C to G in exon 8 or a 13 bp insertion in exon 9 resulted in the termination of the *CmAPRR2* translation [21,23]. In wax gourd, a two-base (GA) insertion in the coding region of *BhAPRR2* (*Bch05G003950*) leads to a change in pericarp color from white to green [24]. In bitter gourd, Matsumura et al. [9] identified the top 15 genes enriched for the uppermost 0.1% SNPs with the highest color association, among which *APRR2* was included. Furthermore, *McAPRR2* was reported to regulate green stigma in bitter gourd [25]. *APRR2* is a transcription factor, and a previous study suggested that it regulates plastid development and maturation in tomatoes [26]. Currently, the genes controlling the pericarp color of the bitter gourd remain unknown, and there is a need to identify the key gene that regulates pericarp color.

This study identified the key gene *McAPRR2* that regulates pericarp color in bitter gourd via a genome-wide association study (GWAS). Our analysis revealed that a 15 bp sequence insertion in the *McAPRR2* gene caused premature termination of the encoded protein sequence. Three haplotypes were identified in *McAPRR2*, and the haplotype of *McAPRR2* was highly correlated with pericarp color and ecological type. Interestingly, this gene was located within a genomic region impacted by domestication. Our findings provide new insight that pericarp color of the bitter gourd was subjected to domestication, and this discovery will enrich the molecular breeding of bitter gourd towards breeders.

## 2. Results

### 2.1. Genetic Characteristics of 106 Bitter Gourd Accessions

**Figure 1:**
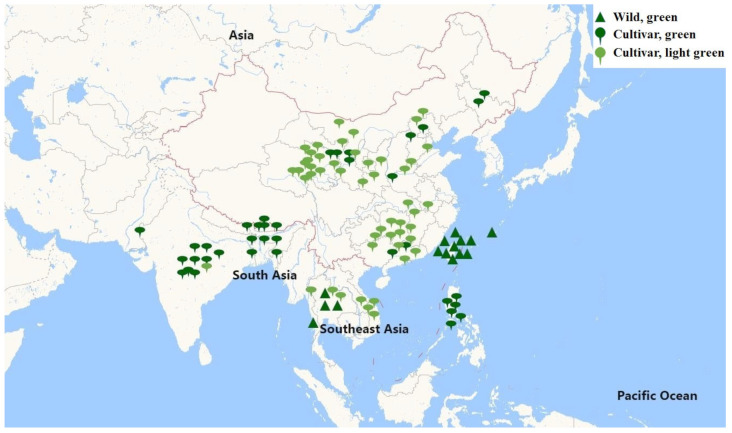
The collection of 106 lines used in this study is widely distributed in Asia. The green triangle represents the wild bitter gourd with green skin. In contrast, cultivated bitter gourds, differentiated by their green and light green skin, are, respectively, denoted by the green and light green droplet icons.

A total of 47 *M. charantia* accessions were collected from China, with one exception. The resequencing of these accessions produced 222.90 Gb of clean data, corresponding to a genomic coverage ranging from approximately 13.12× (KG19) to 19.07× (KG21) (Table S1). For comparative purposes with individuals from distinct geographical regions, an additional 60 *M. charantia* accessions were downloaded from the NCBI database. Therefore, this study included a total of 66 accessions from Southeast Asia (SEA), 24 from South Asia (SA), and 16 from Taiwan (TAI) and Thailand (THAI), along with one outgroup (Figure 1). By mapping the clean reads onto the OHB3-1 reference genome, this study identified a final set of 3,335,160 high-quality SNPs based on the resequencing of 106 accessions.

**Figure 2:**
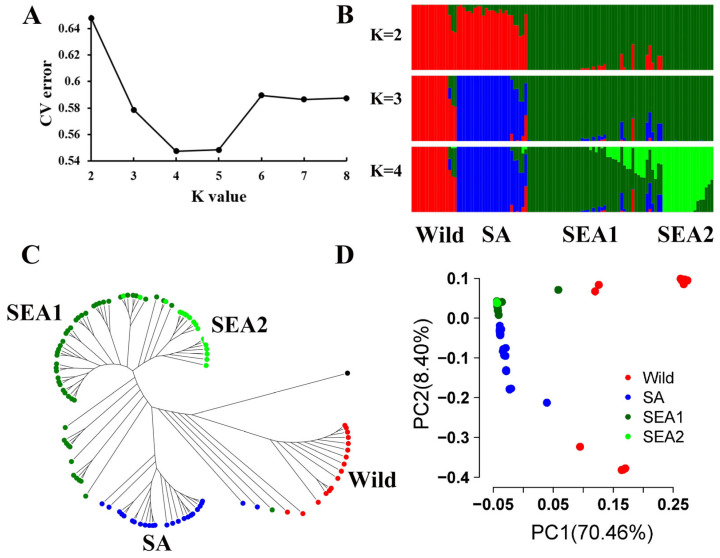
Determination of the genetic structure in 106 bitter gourd accessions. (**A**) ADMIXTURE cross-validation errors for K from 2 to 8. (**B**) Population structure by ADMIXTURE. (**C**) Neighbor-joining tree. (**D**) Principal component analysis. Wild, SA, SEA1, and SEA2 were colored red, blue, dark green, and green, respectively.

To infer the genetic structure of the 106 bitter gourd accessions, ADMIXTURE analysis, phylogenetic tree construction, and principal component analysis (PCA) were employed. Interestingly, our bitter gourd accessions formed groups distinct from the previously published TAI, THAI, SA, and SEA [9]. The 106 accessions in this study clustered into four distinct groups: wild type from TAI and THAI, SA, SEA1, and SEA2 (Figure 2A,B and Table S1). The SEA group initially diverged from the others when K = 2; subsequently, the SA group separated from the wild type when K = 3. When K = 4, SEA split into SEA1 and SEA2, reported for the first time here. Accordingly, the ADMIXTURE models with K = 4 yielded the lowest cross-validation errors (Figure 2A). The neighbor-joining (NJ) tree and PCA were also largely consistent with the ADMIXTURE analysis (Figure 2C,D). PC1 and PC2 explained 70.46% and 8.40% of the variation, respectively. Eight individuals were found to be admixtures among wild, SA, and SEA for max Q < 0.70, while 15 were admixtures between SEA1 and SEA2 (Figure 2B–D and Table S1).

Genome-wide nucleotide diversity (π) for each group was calculated using 3,335,160 SNPs. The wild group displayed a significantly higher π (2.61 × 10^−3^) than the total cultivated bitter gourd groups (0.9 × 10^−3^). Among the cultivated groups, the SA group exhibited the highest π (1.28 × 10^−3^), while the SEA2 group showed the lowest (0.37 × 10^−3^) (Figure S1A). Additionally, the π was relatively consistent across the 11 chromosomes in the wild group, SEA1, and SEA2, except for Chr4 of the SA group, which exhibited higher diversity (Figure S1B). Evaluations of the fixation index value (*Fst*) revealed that SEA1 and SEA2 displayed higher differentiation from the wild group than the SA group (Table S2). SEA1 only weakly differentiated from SEA2, while differentiation between SEA and the SA group was moderate.

### 2.2. Genome-Wide Association of Pericarp Color in Bitter Gourd

**Figure 3:**
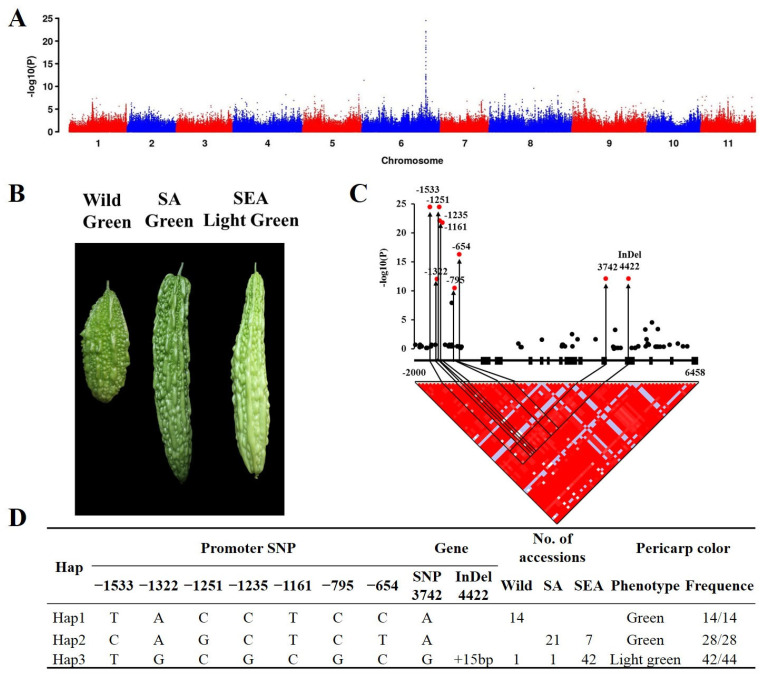
Natural variations in *McAPRR2* were significantly associated with pericarp color in bitter gourd. (**A**) Genome-wide association study (GWAS) of pericarp color across 11 chromosomes. (**B**) The representative pericarp colors of individuals from wild, SA, and SEA groups. (**C**) *McAPRR2*-based association mapping and pairwise LD analysis. Dots indicate SNPs or Indels. The significant SNPs and one Indel (*p* < 1 × 10^−10^) located in the promoter and exon region of *McAPRR2* are marked in red and linked to the pairwise LD diagram with a solid line. Black lines in the pairwise LD diagram emphasize the strong LD of Indel 4422 with the significant variants. (**D**) A haplotype analysis of *McAPRR2* in 86 bitter gourd germplasm accessions.

Significant variation in pericarp color was observed among the 106 bitter gourd accessions from different genetic groups. The wild group typically showed green or dark green pericarp, whereas the cultivated group exhibited a wide array of green shades (Table S1). Specifically, 83.33% of individuals from the SA group displayed medium to dark green pericarp, while 71.21% of individuals from the SEA group exhibited light green or white pericarp (Figure 3B).

A genome-wide association study (GWAS) for pericarp color was conducted using the SNP variations derived from the resequencing of 106 accessions. The GWAS analysis revealed a single peak across the 11 chromosomes (Figure 3A). A total of 41 significantly associated SNPs in Chr6 were identified for pericarp color at the threshold of −log(p) = 10, thereby narrowing down the region to 17.3 kb (Figure 3A,B and Table S3). The quantile–quantile plot demonstrated a deviation of observed values from the expected X = Y line, verifying the reliability of the identified significant association SNPs (Figure S2). Within the candidate region, only one gene, designated as evm.TU.chr6.3557, was located in the OHB3-1 V2 genome. This gene (*McAPRR2*) encodes a two-component response regulator-like protein *APRR2*, which was previously reported to be involved in regulating pericarp color in *Cucurbitaceae* [20,21,23,24].

### 2.3. The Haplotype of McAPRR2

Seven SNPs on the promoter of *McAPRR2*, one nonsynonymous mutation on exon9, and one InDel on exon10 were found to be significantly associated with pericarp color (−log(p) > 10) (Figure 3C). Linkage disequilibrium (LD) analysis of *McAPRR2* revealed a strong LD block between InDel4422 and other significant variants (Figure 3C). Using these significant variants, a haplotype analysis was conducted on 106 bitter gourd accessions. After excluding missing data and heterozygous loci, haplotypes were finally assigned for 86 bitter gourd accessions. These 86 accessions were divided into three haplotypes, namely Hap1, Hap2, and Hap3 (Figure 3D). Hap1, consisting of 14 accessions, exclusively exhibited a green pericarp phenotype and originated entirely from the wild group. Hap2, which included 28 accessions, presented a uniformly green pericarp phenotype, with 75% of samples from the SA group and 25% from the SEA group. Hap3 contained 44 accessions, 95.45% of which displayed a light green pericarp phenotype, primarily originating from the SEA group. Interestingly, one wild type in Hap3 may be the original source of Hap3. Taken together, these findings suggest that the *McAPRR2* haplotype is strongly correlated with pericarp color and geographical location.

### 2.4. The Expression Pattern of McAPRR2 and Pigment Content in Pericarp Color

**Figure 4:**
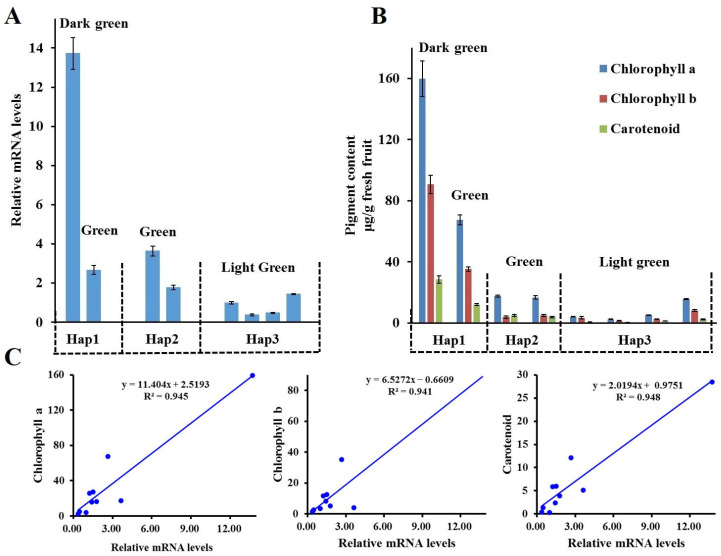
Correlation between *McAPRR2* expression level and pigment content across different haplotypes. (**A**) Expression analysis of *McAPRR2* in Hap1, Hap2, and Hap3. (**B**) Quantification of chlorophyll a, chlorophyll b, and carotenoid pigments in individuals from the three *McAPRR2* haplotypes. (**C**) Direct linear association between *McAPRR2* expression level and pigment content.

The haplotypes of *McAPRR2* were identified in six accessions via Sanger sequencing. The *McAPRR2* in KG113 and KG136 is Hap1, *McAPRR2* in KG19 and KG29 is Hap2, and *McAPRR2* in KG27 and KG44 is Hap3. The expression pattern of *McAPRR2* and pigment content of the three haplotypes were analyzed. *McAPRR2* expression in Hap1 and Hap2 was higher than that in Hap3, with dark green pericarp showing significantly higher *McAPRR2* expression compared to green and light green pericarp (Figure 4A). Additionally, the samples used for analyzing haplotype expression were also used for pigment content assessment. The contents of chlorophyll a, chlorophyll b, and carotenoids in Hap1 were higher than those in Hap2 and Hap3, with Hap2 displaying higher pigment content than Hap3 (Figure 4B). Dark green pericarp exhibited higher pigment content than the other pericarp types. The relationship between *McAPRR2* expression and the levels of chlorophyll a, chlorophyll b, and carotenoids was analyzed, revealing a positive correlation with a coefficient exceeding 0.94 (Figure 4C).

### 2.5. The 15 bp Insertion in McAPRR2 Alters Its Function

**Figure 5:**
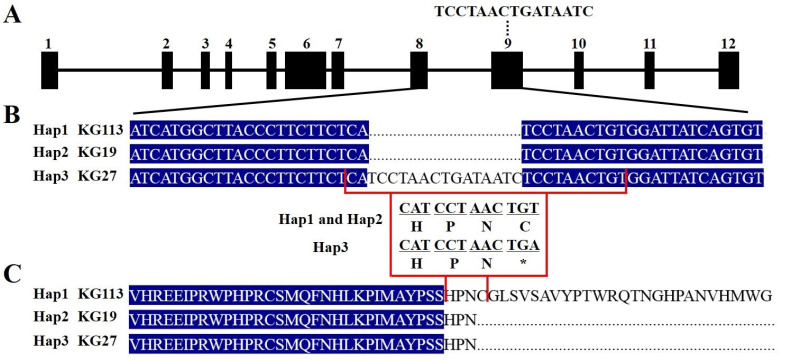
A 15 bp insertion in Hap3 leads to a premature stop codon of McAPRR2. (**A**) Gene structure. InDel4422, a 15 bp insertion, is located in exon9 of *McAPRR2*. (**B**) The alignment of coding sequences (CDS) for Hap1, Hap2, and Hap3 of *McAPRR2*. (**C**) InDel4422 in Hap3 results in a premature stop codon of *McAPRR2*. * represents stop codon.

Sequencing of the *McAPRR2* CDS from the three haplotypes confirmed the existence of SNP3742 and InDel4422 (Figure 5A,B). Notably, in InDel4422, a 15 bp insertion (TCCTAACTGATAATC) in exon 10 of *McAPRR2* leads to a premature termination in the protein encoded by Hap3, inducing a shift in pericarp color from green to light green.

The conserved domains of *McAPRR2* were predicted using InterPro, identifying the Response Regulatory Domain and the Myb Domain. The Response Regulatory Domain is located before the Myb Domain (Figure S3). InDel4422 is not located within either domain. Thus, its mutation does not impact the function of the two domains in *McAPRR2*. *APRR2* was previously reported to significantly influence the pericarp color of watermelon, cucumber, melon, and wax gourd [20,21,24]. In this study, a multiple-sequence alignment of the APRR2 protein sequence was conducted. The mutation sites of these APRR2 proteins are located after the Myb Domain, suggesting they do not affect the function of the two domains, with the exception of the mutation sites in white wax gourd, which are found before the Myb Domain (Figure S3).

### 2.6. McAPRR2 Was under Domestication in Bitter Gourd

**Figure 6:**
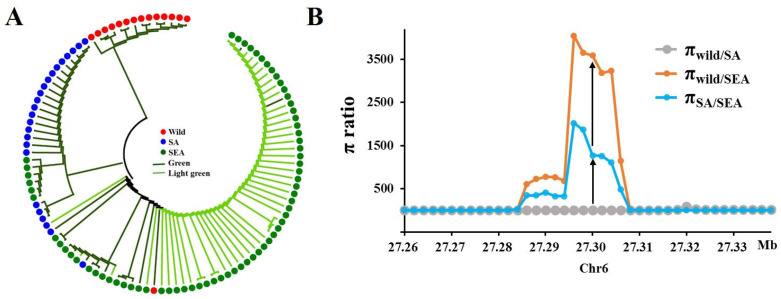
The *McAPRR2* gene has undergone domestication. (**A**) Phylogenetic tree constructed based on 74 SNPs in the *McAPRR2* gene region and promoter. The tree is clustered according to pericarp color and genetic group. (**B**) Analysis of nucleotide diversity in genetic group. Low π_wild/SA_ values and high π_wild/SEA_ and π_SA/SEA_ values indicate that pericarp color was domesticated in SEA.

*McAPRR2* haplotype is significantly correlated with both pericarp color and ecological type. Hap1 was strictly associated with wild types exhibiting green pericarp, while Hap2 corresponded to SA and SEA types with green pericarp. Hap3 was mainly related to SEA types, distinguished by a light green pericarp. The insertion of a 15 bp sequence in Hap3 altered the function of *McAPRR2*, leading to a modification in pericarp color. These findings suggest that *McAPRR2* was potentially subjected to selection during domestication. To investigate whether *McAPRR2* was subject to domestication selection, the phylogenetic tree and nucleotide diversity of this gene were analyzed. Using 74 SNPs located in the *McAPRR2* gene region and its promoter, a phylogenetic tree was constructed. The tree generally clustered according to pericarp color and genetic group (Figure 6A). Green pericarp types were clustered together, as were light green pericarp types. Wild types were clustered together, while SA and a subset of green pericarp SEA types formed another group, and SEA types were gathered in yet another cluster. The nucleotide diversity in the *McAPRR2* gene showed that the π_wild/SA_ value was the lowest, indicating low nucleotide diversity between these two groups on the *McAPRR2* gene (Figure 6B). High π_wild/SEA_ and π_SA/SEA_ values indicate high nucleotide diversity between wild and SEA types and between SA and SEA types on the *McAPRR2* gene (Figure 6B). The loss of genome-wide genetic diversity in modern crops is a typical characteristic of plant domestication. In conjunction with haplotype and pericarp color phenotypes, this study concluded that pericarp color underwent domestication in the SEA region, leading to the formation of the light green pericarp phenotype.

## 3. Discussion

The current study resequenced 47 accessions and incorporated the resequencing data of an additional 60 accessions as reported by Matsumura et al. [9]. In this study, we classified 106 bitter gourds into groups: a wild group consisting of TAI and THAI, SA, SEA1, and SEA2. This is the first study to propose that the SEA group could be subdivided into SEA1 and SEA2 (Figure 2). SEA1 was only weakly differentiated from SEA2 (Fst = 0.02), making it difficult to detect the structure for the previously reported 19 resequenced accessions of the SEA group [9]. Cui et al. [10] detected SA, SEA, China, and Tanzania groups in a set of 166 germplasms. The accession from China was also SEA [10], and this study classified SEA into two groups; this result is consistent.

We identified a significant 17.3 kb region that is highly associated with pericarp color via GWAS, which has been extensively utilized in many plants and has proven to be an effective method for identifying crucial genes for various traits [23,27,28,29]. Interestingly, *McAPRR2* was the only gene located in this significant region. In bitter gourd, Matsumura et al. [9] identified the top 15 genes that were enriched for the top 0.1% of SNPs with the highest color association, and *APRR2* was among these genes. *APRR2* was the second-to-last gene associated with pericarp color, and they failed to identify the key gene regulating pericarp color due to an inadequate sample size for the GWAS. Additionally, *McAPRR2* was reported to regulate green stigma in bitter gourd [25]. Taken together with our study, *McAPRR2* regulates pericarp color and stigma color in bitter gourd, which is consistent with *CsAPRR2* in cucumbers that *CsAPRR2* regulates pericarp color, stem, leaf, and flower [20]. Furthermore, a 15 bp insertion in exon 10 of *McAPRR2* leads to a premature termination in the protein encoded by *McAPRR2*, which is associated with a light green pericarp (Figure 5). This finding is consistent with studies in *Cucurbitaceae* crops where the premature termination of the APRR2 protein also controls pericarp color, further reinforcing the role of *McAPRR2* in regulating pericarp color in our study [20,21,23,24]. The green pericarp-associated haplotype Hap1 was uniquely observed in the wild group, while Hap2, also associated with green pericarp, was confined to the SA and SEA groups. Hap3, linked with the light green pericarp, predominantly surfaced within the SEA group. Some dark green and green pericarp bitter gourds are the same haplotype. The may factor could affect the pericarp color in bitter gourd, such as the transcription efficiency of APRR2.

Color is a significant trait in plants, and numerous studies have reported that the color of various plant species has undergone domestication and selection processes, such as the berry color of grape [30], pericarp color [15], and seed color of grain [31]. Bitter gourd, primarily cultivated in Asia, serves dual roles as both a vegetable and a significant medicinal crop. However, its limited demand has resulted in a slower rate of domestication compared to other cucurbit crops. The domestication of bitter gourd appears to be a complex process, with particular human preferences varying across different cultures. South Asians, for example, favor highly bitter, smaller fruits (although larger than their wild counterparts) with spiny, dark green features. In contrast, Southeast Asians prefer less bitter fruits with light green (or white) and smooth skin [18]. In this study of 106 bitter gourd accessions, wild types were green or dark green, SA types were mainly dark green, and SEA types were predominantly light green or white. The distribution of bitter gourd pericarp color evidently correlates with geographical location (Figure 1). Existing research suggests that selection pressures during the course of domestication and subsequent selection processes are associated with adaptations to specific geographical locations [16]. Therefore, it is plausible that *McAPRR2* was under domestication and selection pressures. The haplotypes of *McAPRR2* display a high correlation with both pericarp color and ecological type (Figure 3D). The phylogenetic tree of *McAPRR2* clustered according to pericarp color and ecological type (Figure 6A). Moreover, the nucleotide diversity of *McAPRR2* exhibits a gradual decline among the wild, SA, and SEA groups (Figure 6B). These results collectively suggest that the pericarp color of bitter gourd primarily underwent domestication selection.

To date, despite several studies reporting on the evolution of bitter gourd, these findings remain inconsistent, leaving the evolutionary history of bitter gourd inadequately elucidated. It is unequivocally established that bitter gourd originated in Africa [5] and was domesticated in Asia [7,8]. Cui et al. [10] proposed that the bitter gourd was domesticated in South Asia (SA). In contrast, Matsumura et al. [9] reported that the signals of selection between wild and Southeast Asia (SEA) types predominantly arose from the selection during the SA–SEA divergence stage rather than the result of a gradual and accumulative evolution from wild to SA to SEA. Renner [11], integrating insights from two parallel studies, proposed that the bitter gourd expanded from Africa to Southeast Asia and underwent domestication there [6,11]. The domestication selection of bitter gourd pericarp color is associated with consumer preferences; individuals in the SEA region tend to favor a lighter green pericarp color [18]. Considering the results of our study, the pericarp color of bitter gourd underwent domestication in the SEA region, a finding that aligns with Renner’s report. Future studies should include a broader range of accessions from varied geographical locations to more effectively elucidate the history of bitter gourd domestication.

## 4. Materials and Methods

### 4.1. Resequencing of 106 M. charantia Accessions

A total of 106 accessions of *M. charantia* were used in this study. The resequencing data of 60 accessions were obtained from the NCBI BioProject PRJNA578358 with one outgroup [9], while the remaining 47 accessions were resequenced in house (Table S1). Among the 47 accessions, 46 were collected from China. The 47 accessions were germinated at a constant temperature of 30 °C and grown in an artificial climate chamber. Genomic DNA was extracted from young leaves using the NuClean Plant Genomic DNA Kit. Paired-end (PE) libraries with an insert size of approximately 350 bp were constructed for each accession, and sequencing was performed on the Novaseq platform with PE 2 × 150 bp.

### 4.2. The SNP Calling and Filtering

The raw reads were trimmed for sequence quality and adaptor removal using Trimmomatic with the parameters “SLIDINGWINDOW:4:20 LEADING:3 TRAILING:3 MINLEN:40” [32]. The resulting clean reads were aligned to the OHB3-1 V2 genome using the MEM algorithm implemented in the Burrows –Wheeler Alignment Tool (BWA) [33]. PCR duplicated reads were removed using Picard (https://broadinstitute.github.io/picard/, accessed on 8 October 2018). SNP genotypes were called using the HaplotypeCaller module of the Genome Analysis Toolkit (GATK) and were filtered according to the GATK Best Practices recommendations [34]. High-quality (HQ) SNPs were obtained by selecting biallelic SNP sites with a QUAL score > 30, a missing rate < 10%, and a minor allele frequency (MAF) > 5% using vcftools V0.1.15 [35]. A total of 3,335,160 SNPs passed the filtering criteria.

### 4.3. Population Genetics Analyses

The 3,335,160 SNPs were pruned to 68,832 SNPs using the “--indep-pairwise 50 5 0.5” command in Plink V1.07. The pruned SNPs were then used to detect population structure using ADMIXTURE V1.3.0 with K values ranging from 2 to 8 [36,37]. A subset of 74,616 SNPs located at four-fold degenerate sites from the HQ SNPs was used to construct a neighbor-joining (NJ) tree in MEGA [38]. Principal Component Analysis (PCA) was performed using all 3,335,160 SNPs in EIGENSOFT. Nucleotide diversity (π) and pairwise *Fst* were calculated in 50 kb windows with 10 kb steps using vcftools [35].

### 4.4. Genome-Wide Association (GWAS) of Pericarp Color

The pericarp color of 60 accessions was measured with white, light green, medium green, green, and dark green [9]. In order to make use of the resequencing data of the 60 accessions and to identify the key gene regulating the pericarp color, the pericarp color was also categorized into five groups: white, light green, medium green, green, and dark green. The 47 accessions were planted in Tongzhou (Beijing) from 2020 to 2022. The pericarp color was measured after 10 days after pollination and compared to the color in Figure 3B described in Matsumura et al. [9]. GWAS was performed using a total of 3,335,160 SNPs that passed the filtering criteria: biallelic SNP sites, QUAL > 30, missing rate < 10%, and MAF > 5%. PCA was employed to correct for population stratification, and GWAS analyses were conducted using the GLM algorithm in TASSEL [39].

### 4.5. The Genetic Analysis of McAPRR2 Gene

The *McAPRR2* haplotype was determined using the Haploview V4.2 software based on seven highly related SNPs in the *McAPRR2* promoter region, as well as two functional mutations in the coding sequence (CDS) of *McAPRR2* [40]. A phylogenetic tree was constructed in MEGA using 74 SNPs located in the promoter and gene region of *McAPRR2* [38]. The π of the wild, SA, and SEA groups was calculated in 10 kb windows with 2 kb steps using vcftools [35]. The *APRR2* protein sequence, previously reported to regulate pericarp color within the *Cucurbitaceae* family, was used in a multiple sequence alignment performed with DNAMAN.

### 4.6. RT-qPCR Analysis of McAPRR2

Pericarp tissues from six different accessions, namely KG113, KG136, KG19, KG29, KG27, and KG44, with distinct haplotypes were utilized for qRT-PCR analysis. Sanger sequencing was employed to sequence the *McAPRR2* of all six accessions. Specifically, Hap1 was identified in KG113 and KG136, Hap2 in KG19 and KG29, and Hap3 in KG27 and KG44. RNA extraction from the pericarp at 10 days post-pollination was carried out using the Plant Total RNA Extraction Kit (Vazyme, Nanjing, China, RC411) following the manufacturer’s instructions. The *McAPRR2* coding sequence (CDS) was subsequently amplified from the total RNA using the primers listed in Table S4. First-strand cDNA was synthesized using the First Strand cDNA Synthesis Kit (Vazyme, Nanjing, China, R223), with 1 μg of total RNA. The qRT-PCR reaction was performed in a total volume of 10 μL, consisting of 1 μL of cDNA template, 0.2 μL of each primer (10 μM), 5 μL of TB Green Premix Ex Taq (2×, TaKaRa, Beijing, China, RR460), and 3.6 μL of RNase-free water. The PCR conditions were as follows: initial denaturation at 95 °C for 30 s, followed by 40 cycles of denaturation at 95 °C for 5 s and annealing/extension at 60 °C for 34 s; and then, a final melting curve step of 95 °C for 15 s, 60 °C for 1 min, and 95 °C for 15 s. qRT-PCR was conducted on the LightCycler 480 System (Roche Diagnostics, Basel, Switzerland). The relative expression levels were computed using the comparative Ct method (2^−ΔΔCt^) [41]. The primers employed for qRT-PCR are listed in Table S4.

### 4.7. Analysis and Quantification of Chlorophyll and Carotenoid Contents

The sample used for pigment content measurement was the same as that used for qRT-PCR. The chlorophyll and carotenoid contents of the pericarp were determined using spectrophotometry that described in the previous study [24].

## Data Availability

The raw data have been uploaded to the GSA (https://bigd.big.ac.cn/gsa/, accessed on 8 October 2018) under the accession number CRA011965.

## Supplementary Materials

The following supporting information can be downloaded at https://www.mdpi.com/article/10.3390/plants12203585/s1. Figure S1: Nucleotide diversity comparison among different genetic groups. (A) Comparison of nucleotide diversity within wild, SA, SEA1, and SEA2 populations. (B) Nucleotide diversity across 11 chromosomes in wild, SA, SEA1, and SEA2. Figure S2: Quantile-Quantile plot of genome-wide association study (GWAS) analysis of pericarp color. Figure S3: Multi-sequence alignment based on APRR2 protein sequence in the *Cucurbitaceae*. *APRR2* contains both the Response regulatory domain and the Myb domain. The alteration in fruit skin color is due to the premature termination of the protein encoded by *APRR2*. Table S1: Sequencing Summary for 47 *M. charantia* accessions. Table S2: Pairwise *Fst* comparisons among the genetic groups. Table S3: Significant pericarp color-associated SNPs identified via GWAS. Table S4: Primer used in this study.
