# Supplementary material for: McAPRR2: The Key Regulator of Domesticated Pericarp Color in Bitter Gourd
Source: Plants (Basel). 2023 Oct 16;12(20):3585. doi: 10.3390/plants12203585 (PMC10610206; doi:10.3390/plants12203585)
Supplement: Supplementary file 1 [file plants-12-03585-s001.zip › plants-2642204-supplementary.pdf]

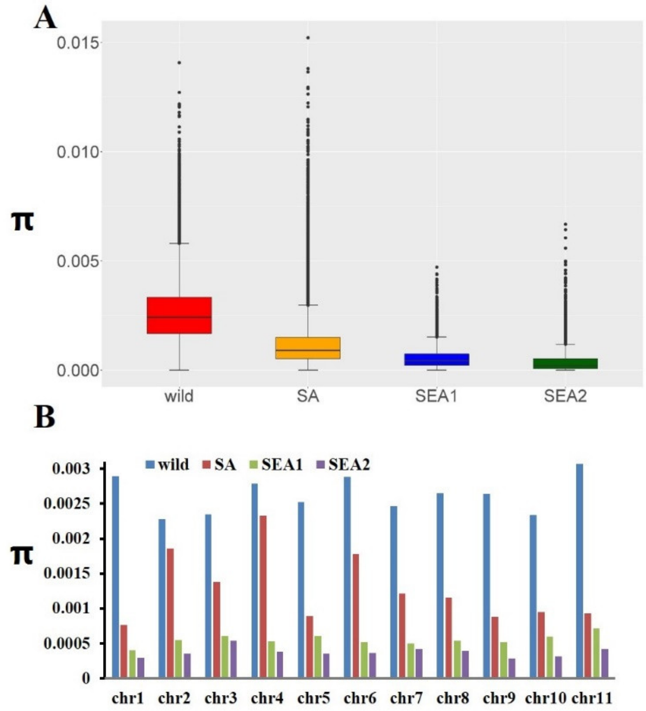

Figure S1. Nucleotide diversity comparison among different genetic groups. (A) Comparison of nucleotide diversity within wild, SA, SEA1, and SEA2 populations. (B) Nucleotide diversity across 11 chromosomes in wild, SA, SEA1, and SEA2.

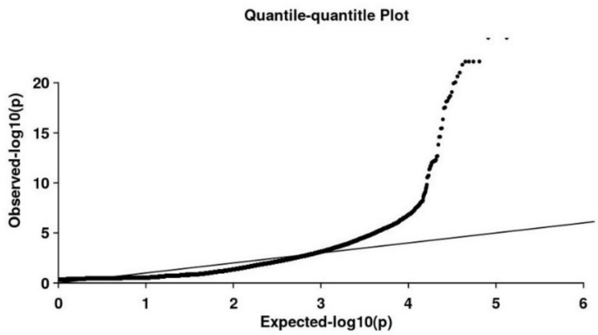

Figure S2. Quantile-Quantile plot of genome-wide association study (GWAS) analysis of pericarp color.

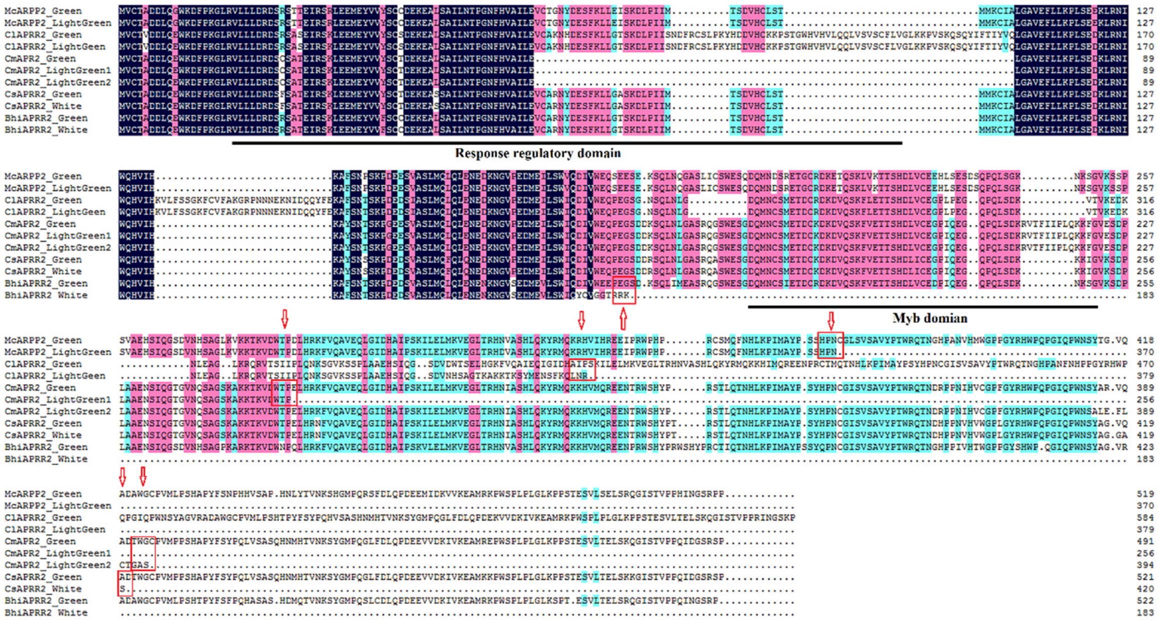

Figure S3. Multi-sequence alignment based on APRR2 protein sequence in the *Cucurbitaceae*. *APRR2* contains both the Response regulatory domain and the Myb domain.

Table S1. Sequencing summary for 47 *M. charantia* accessions.

**ID**	**Name**	**Clean reads (bp)**	**Depth***	**Color**	**Genetic group**	**Admixture**
KG10	Jan-89	5,209,685,252	17.19	Light green	SEA1	N
KG11	Changbai No.1	4,300,659,095	14.19	Light green	SEA1	N
KG13	Xiang	4,590,777,525	15.15	Light green	SEA1	N
KG14	Kan	4,689,050,226	15.48	Green	SEA1	N
KG15	NERCV No.9	4,528,214,073	14.94	Light green	SEA1	Y
KG16	Qingpi	4,267,382,512	14.08	Light green	SEA2	N
KG17	Lvren	4,380,248,163	14.46	Light green	SEA1	Y
KG18	Changlv No.1	4,699,558,314	15.51	Green	SEA1	N
KG19	Changlv No.2	3,975,598,921	13.12	Green	SEA1	N
KG20	NERCV No.10	4,229,886,464	13.96	Light green	SEA1	N
KG21	Hengfeng	5,778,770,561	19.07	Light green	SEA1	N
KG23	Bai	4,856,802,780	16.03	White	SEA1	N
KG24	Yangzizhou	4,593,662,266	15.16	Light green	SEA1	N
KG25	Yuan	4,694,252,976	15.49	Green	SEA1	N
KG26	Changbai	5,745,344,037	18.96	White	SEA1	N
KG27	Tongbai	4,369,213,380	14.42	Light green	SEA1	N
KG28	Xinyang	5,052,238,602	16.67	Light green	SEA1	N
KG29	NERCV No.11	4,182,254,688	13.80	Green	SA	N
KG31	Gongyi	4,537,246,673	14.97	Green	SEA1	N
KG32	Dabai	4,627,846,790	15.27	White	SEA1	N
KG33	Lengjiang	5,334,521,218	17.61	Light green	SEA1	N
KG34	Xingao No.1	4,754,862,067	15.69	Light green	SEA2	N
KG35	Lanshan	5,349,894,559	17.66	White	SEA1	Y
KG36	Cuiyou No.2	5,341,532,970	17.63	Dark green	SEA1	N
KG37	Darou No.2	5,006,215,869	16.52	Light green	SEA2	N
KG38	Jiaqing No.6	4,850,821,784	16.01	Light green	SEA2	N
KG39	NERCV No.1	4,505,888,472	14.87	Light green	SEA1	N
KG40	NERCV No.2	4,894,032,471	16.15	Light green	SEA1	N
KG41	NERCV No.3	5,515,755,746	18.20	Light green	SEA1	N
KG42	NERCV No.4	4,201,646,489	13.87	Green	SEA1	N
KG43	NERCV No.5	4,652,197,245	15.35	Light green	SEA1	Y
KG44	NERCV No.6	4,845,436,237	15.99	Light green	SEA1	N
KG45	NERCV No.7	4,244,683,704	14.01	Light green	SEA1	N
KG46	Liangku1403	4,993,902,241	16.48	Light green	SEA1	N
KG47	Liangku1405	4,425,910,652	14.61	Light green	SEA1	N
KG48	Liangku1406	4,052,142,339	13.37	Light green	SEA2	Y
KG49	Liangku1401	4,414,675,512	14.57	Light green	SEA1	Y
KG50	Gedalv	4,800,192,386	15.84	Green	SEA1	N
KG51	Liangku1402	3,991,013,922	13.17	Light green	SEA2	Y
KG52	Panduola	4,166,535,486	13.75	Light green	SEA1	Y
KG53	Guinongkeyu	5,766,039,438	19.03	Light green	SEA2	N

	No.1					
KG54	Guangliang No.2	4,470,736,491	14.76	Dark green	SEA1	N
KG55	Fenglv	4,533,542,368	14.96	Light green	SEA2	N
KG56	Guangliang No.3	5,369,234,129	17.72	Light green	SEA1	Y
KG57	NERCV No.8	4,964,457,059	16.38	Light green	SEA1	Y
KG58	MC1-6-12	4,979,619,399	16.43	Light green	SEA2	N
KG59	Guinongkeyu No.2	5,162,680,178	17.04	Light green	SEA2	N
SRR10309996	AVBG1656	5,336,644,200	17.61	Green	SA	N
SRR10309997	AVBG1655	5,096,781,900	16.82	Green	SA	N
SRR10309998	AVBG1653	5,711,358,900	18.85	Green	SA	N
SRR10310000	AVBG1325	4,963,381,800	16.38	Green	SA	N
SRR10310001	VI051072	11,074,473,000	36.55	Green	SEA1	Y
SRR10310002	VI050202	17,986,629,000	59.36	Green	wild	N
SRR10310003	VI050201	14,550,581,700	48.02	Green	wild	N
SRR10310004	VI050194	17,124,514,800	56.52	Green	wild	N
SRR10310005	VI050164	14,222,486,100	46.94	Green	wild	N
SRR10310006	VI050146	13,873,333,800	45.79	Green	wild	N
SRR10310007	VI050132	15,541,261,800	51.29	Green	wild	Y
SRR10310008	VI050130	14,436,072,000	47.65	Green	wild	Y
SRR10310009	VI048924	13,048,854,300	43.07	Green	wild	Y
SRR10310010	VI047624	13,209,138,600	43.60	Green	wild	N
SRR10310011	AVBG1322	5,603,495,100	18.49	Green	SA	N
SRR10310012	VI047622	14,259,361,200	47.06	Green	wild	N
SRR10310013	VI039908	13,750,800,600	45.38	Green	SA	Y
SRR10310014	CRL003	13,643,767,800	45.03	Green	wild	N
SRR10310015	CRL002	18,242,415,600	60.21	Green	wild	N
SRR10310016	CRL001	12,088,693,800	39.90	Green	wild	N
SRR10310017	THMC95	5,871,930,300	19.38	Green	SA	N
SRR10310018	THMC629	4,920,527,700	16.24	Light green	SEA2	N
SRR10310019	THMC600	5,133,291,600	16.94	Light green	SEA2	N
SRR10310020	THMC599	4,921,120,200	16.24	Light green	SEA2	N
SRR10310021	THMC594	4,387,076,400	14.48	Light green	SEA2	N
SRR10310022	AVBG1321	6,459,704,700	21.32	Green	SA	N
SRR10310023	THMC549	5,519,676,900	18.22	Green	SEA1	N
SRR10310024	THMC542	5,320,039,200	17.56	Dark green	SEA1	N
SRR10310025	THMC53-2	5,432,392,200	17.93	Green	SEA1	N
SRR10310026	THMC532	5,755,887,900	19.00	Light green	SEA2	N
SRR10310027	THMC522	5,478,624,000	18.08	Light green	SEA2	N
SRR10310028	THMC519	7,101,306,300	23.44	Light green	SEA1	N
SRR10310029	THMC518	6,431,168,400	21.23	Light green	SEA1	N
SRR10310030	THMC483	4,529,352,900	14.95	Green	SA	N

SRR10310031	THMC423	5,976,345,300	19.72	Green	SA	N
SRR10310032	THMC422	4,822,162,500	15.92	Green	SA	N
SRR10310033	AVBG1313	5,786,489,400	19.10	Light green	SEA2	N
SRR10310034	THMC421	5,132,457,000	16.94	Green	SA	N
SRR10310035	THMC42	5,284,981,200	17.44	Medium green	SEA1	N
SRR10310036	THMC406	5,661,171,900	18.68	Light green	SEA1	N
SRR10310037	THMC378	5,558,019,900	18.34	Light green	SA	N
SRR10310038	THMC370	5,618,973,000	18.54	Light green	SEA1	N
SRR10310039	THMC348	4,994,664,300	16.48	Green	SA	N
SRR10310040	THMC33	5,711,089,800	18.85	Dark green	SEA1	N
SRR10310041	THMC30	6,411,795,300	21.16	Green	SEA1	N
SRR10310042	THMC219	5,534,163,000	18.27	Green	SA	N
SRR10310043	THMC179	5,239,254,600	17.29	White	SA	N
SRR10310044	AVBG1311	5,431,794,300	17.93	Light green	SEA2	N
SRR10310045	THMC170	5,454,806,100	18.00	Green	wild	N
SRR10310046	THMC167	4,494,262,500	14.83	Dark green	SA	N
SRR10310047	THMC156	4,897,944,900	16.17	Green	SA	N
SRR10310048	THMC153	7,086,205,200	23.39	Green	wild	N
SRR10310049	THMC149	7,017,816,000	23.16	Green	SA	N
SRR10310050	THMC145	5,285,817,900	17.45	Medium green	SA	N
SRR10310051	THMC144	4,487,121,600	14.81	Green	SA	N
SRR10310052	THMC143	5,077,317,300	16.76	Medium green	SA	N
SRR10310053	THMC113	5,359,237,200	17.69	Green	wild	N
SRR10310054	THMC 105-1-1	6,330,073,800	20.89	Green	SA	N
SRR10310055	AVBG1308	5,377,929,300	17.75	Medium green	SEA1	N

Table S2. Pairwise *Fst* comparisons among the genetic groups.

**Group**	**SA**	**SEA1**	**SEA2**
**wild**	0.51	0.61	0.6
**SA**		0.11	0.13
**SEA1**			0.02

Table S3. Significant pericarp color-associated SNPs identified through GWAS.

Chromosome	Pos	-log（P)
chr6	27295224	14.58981347
chr6	27295812	12.64206515
chr6	27295890	12.68020711
chr6	27296174	11.90893127
chr6	27296380	12.19621524
chr6	27296447	12.19621524
chr6	27296544	11.70958694
chr6	27296674	14.59904777
chr6	27297065	13.8158198
chr6	27297120	10.7101436
chr6	27297142	11.42495078
chr6	27297776	12.31661071
chr6	27297951	10.72939103
chr6	27298114	12.19621524
chr6	27298245	11.65183148
chr6	27298454	12.06326012
chr6	27301330	10.58387563
chr6	27304059	17.47180344
chr6	27304165	18.12089265
chr6	27304199	15.43527477
chr6	27304449	15.45531198
chr6	27304666	17.53659979
chr6	27305646	19.93531743
chr6	27305664	18.48748904
chr6	27305726	20.6298016
chr6	27305805	18.68141499
chr6	27305846	18.18950537
chr6	27305916	19.07236278
chr6	27306119	22.11940933
chr6	27306304	22.11940933
chr6	27306441	20.05146478
chr6	27306450	22.11940933
chr6	27306665	21.00493107
chr6	27307230	24.49555632
chr6	27307441	12.08186477
chr6	27307512	24.49555632
chr6	27307528	22.13151426
chr6	27307602	21.80779507
chr6	27307968	10.53884677
chr6	27308109	16.34691297
chr6	27312505	12.11570887

Table S4. Primer used in this study.

Primer ID		Primers(5'-3')	Purpose
McAPRR2_1	Forward	CCAAGAAGAGGCGAAAACATGACC	Primer used to amplification of *McAPRR2* coding region
	Reverse	TATTTAATAAACATTATACAGACAGAGGG TGTGG	
McAPRR2_2	Forward	TGTTGTTGGCCTTGTGACTG	Primers used for qRT-PCR analysis of *McAPRR2*
	Reverse	CCCAATCGGATAAGCCTCAG	
Mc18SDL1F	Forward	ACCGAACCCATGTTCACTC	Reference gene for *McAPRR2* qRT-PCR analysis
	Reverse	CAAGCACGGAAGAAAATCGAA	
